# Comparison of Multimodal Deep Learning Approaches for Predicting Clinical Deterioration in Ward Patients: Observational Cohort Study

**DOI:** 10.2196/75340

**Published:** 2025-06-11

**Authors:** Charles A Kotula, Jennie Martin, Kyle A Carey, Dana P Edelson, Dmitriy Dligach, Anoop Mayampurath, Majid Afshar, Matthew M Churpek

**Affiliations:** 1 Department of Medicine, University of Wisconsin–Madison, 610 Walnut St, Madison, WI, 53792, United States, 1 608-262-9564; 2 Department of Medicine, University of Chicago, Chicago, IL, United States; 3 Department of Computer Science, Loyola University Chicago, Chicago, IL, United States; 4 Department of Biostatistics and Medical Informatics, University of Wisconsin–Madison, Madison, WI, United States

**Keywords:** clinical deterioration, deep learning, time series, artificial intelligence, machine learning

## Abstract

**Background:**

Implementing machine learning models to identify clinical deterioration in the wards is associated with decreased morbidity and mortality. However, these models have high false positive rates and only use structured data.

**Objective:**

We aimed to compare models with and without information from clinical notes for predicting deterioration.

**Methods:**

Adults admitted to the wards at the University of Chicago (development cohort) and University of Wisconsin-Madison (external validation cohort) were included. Predictors consisted of structured and unstructured variables extracted from notes as concept unique identifiers (CUIs). We parameterized CUIs in 5 ways: standard tokenization (ST), *International Classification of Diseases* rollup using tokenization (ICDR-T), *International Classification of Diseases* rollup using binary variables (ICDR-BV), concept unique identifiers as SapBERT embedding (SE), and concept unique identifier clustering using SapBERT embeddings (CC). Each parameterization method combined with structured data and each structured data-only method were compared for predicting intensive care unit transfer or death in the next 24 hours using deep recurrent neural networks.

**Results:**

The development (University of Chicago) cohort included 284,302 patients, while the external validation (University of Wisconsin-Madison) cohort included 248,055 patients. In total, 4.9% (n=26,281) of patients experienced the outcome. The SE model achieved the highest area under the precision-recall curve (0.208), followed by CC (0.199) and the structured-only model (0.199), ICDR-BV (0.194), ICDR-T (0.166), and ST (0.158). The CC and structured-only models achieved the highest area under the receiver operating characteristic (0.870), followed by ICDR-T (0.867), ICDR-BV (0.866), ST (0.860), and SE (0.859). Regarding sensitivity and positive predictive value, the CC model achieved the greatest positive predictive value (12.53%) and sensitivity (52.15%) at the cutoff that flagged 5% of the observations in the test set. At the 15% cutoff, the ICDR-T, CC, and ICDR-BV models tied for the highest positive predictive value at 5.67%, while their sensitivities were 70.95%, 70.92%, and 70.86%, respectively. All models were well calibrated, achieving Brier scores in the range of 0.011‐0.012. The modified integrated gradients method revealed that CUIs corresponding to terms such as “NPO – nothing by mouth,” “chemotherapy,” “transplanted tissue,” and “dialysis procedure” were most predictive of deterioration.

**Conclusions:**

A multimodal model combining structured data with embeddings using SapBERT had the highest area under the precision-recall curve, but performance was similar between models with and without CUIs. Although the addition of CUIs from notes to structured data did not meaningfully improve model performance for predicting clinical deterioration, models using CUIs could provide clinicians with relevant information and additional clinical context for supporting decision-making.

## Introduction

### Background

Clinical deterioration, defined as “an acute worsening of a patient’s clinical status that poses a substantial increase to an individual’s short-term risk of death or serious harm,” occurs in up to 5% of hospitalized patients [1,2]. Early detection of clinical deterioration is essential for minimizing preventable death, as delayed intensive care unit (ICU) transfers and rapid response team activations lead to increased morbidity and mortality [3-6]. Current methods of early detection range from simple rule-based tools to complex machine learning models. Prior research has shown that machine learning models often achieve superior performance compared to commonly used rule-based tools, such as the Modified Early Warning Score and the National Early Warning Score [1,7-11]. More importantly, using machine learning models for clinical deterioration as decision support tools in real-world hospital settings has been associated with decreased mortality [12,13]. However, such models still have room for improvement.

Although machine-learning-based early warning scores are more accurate than simpler scores, they still endure high false positive rates (FPR), and their input is often restricted to structured electronic health record (EHR) variables such as vital signs and laboratory results [1,11]. Consequently, most EHR data, which are unstructured, are not used for predicting clinical deterioration. The unstructured component of EHR data includes items such as clinical notes, radiology images, and pathology reports, all of which may contain valuable information for health care providers when it comes time to make decisions. Among these unstructured data types, clinical notes may be especially amenable to enhancing the accuracy of models for predicting deterioration because they contain information about comorbidities, acute medical conditions, and other important risk factors. Therefore, incorporating information from unstructured clinical notes could improve model accuracy, including decreasing the FPR, and provide additional clinical context for clinicians by highlighting medical terms that increase deterioration risk. To accomplish this, the information from the structured data and the clinical notes could be combined and used in what is referred to as a multimodal model, a model that uses data from multiple input types. Several methods of combining information from structured data and clinical notes have been shown to enhance performance in other medical domains [14-21]. However, it remains unknown which approach works best when predicting clinical deterioration in hospitalized patients outside the ICU.

### Objective

To address the high FPR and unimodality of most deterioration models, we aim to compare the performance of multimodal deep learning models that use information from unstructured clinical notes extracted in the form of concept unique identifiers (CUIs)—strings of text from clinical notes that map to medical terms—for use in multimodal modeling of clinical deterioration. The use of CUIs may increase the amount of medically relevant information available to a model at prediction time, as well as improve the overall performance and clinical utility of the deep learning deterioration models. It could also add important clinical context regarding potential risk factors and the cause of clinical deterioration, increasing their value as decision support tools. Lastly, we aim to compare these methods to a model that uses only structured data to determine if incorporating information from clinical notes improves model performance.

## Methods

### Patient Population and Data Collection

Adults (age ≥18 years) admitted to the medical-surgical wards at the University of Chicago (UC) from 2016 to 2022 and the University of Wisconsin-Madison (UW) from 2009 to 2020 with clinical notes data were eligible for inclusion. Our primary outcome was clinical deterioration, defined as death or direct ward to ICU transfer within 24 hours of each observation, with UC used for model development and UW for external validation.

Patient data were retrieved from the enterprise data warehouses of each university’s health system, which included demographics, vital signs, laboratory values, and clinical notes. All data from UC were deidentified in accordance with the Health Insurance Portability and Accountability Act and transferred to UW for analysis. This study was approved by the institutional review boards of UW (#2019‐1258) and UC (#18‐0447).

### Structured Features

Fifty-five structured predictor variables were used to develop our models, including demographics, vital signs, laboratory results, and nurse documentation (Table S1 in Multimedia Appendix 1). For most predictor variables, missing values were imputed by carrying forward in time the last known value, and the remaining missing values were imputed in the development and external validation cohorts using the variable’s median value across all patients in the development cohort. The last known value was only carried forward 24 hours before their missing values were imputed for lactate and blood gas laboratory tests. By using strictly only current and past values when developing and testing the models, as well as a deep learning architecture that only considers these data and not future data, data leakage was minimized. Piecewise-linear encoding transformation, a method demonstrated to improve the performance of deep learning models in large, numerical datasets, was used to create a higher-dimensional representation of the data [22]. An additional “hours since admission” input feature was created to capture temporal information.

### Unstructured Input Parameterization

#### Overview

**Figure 1.**
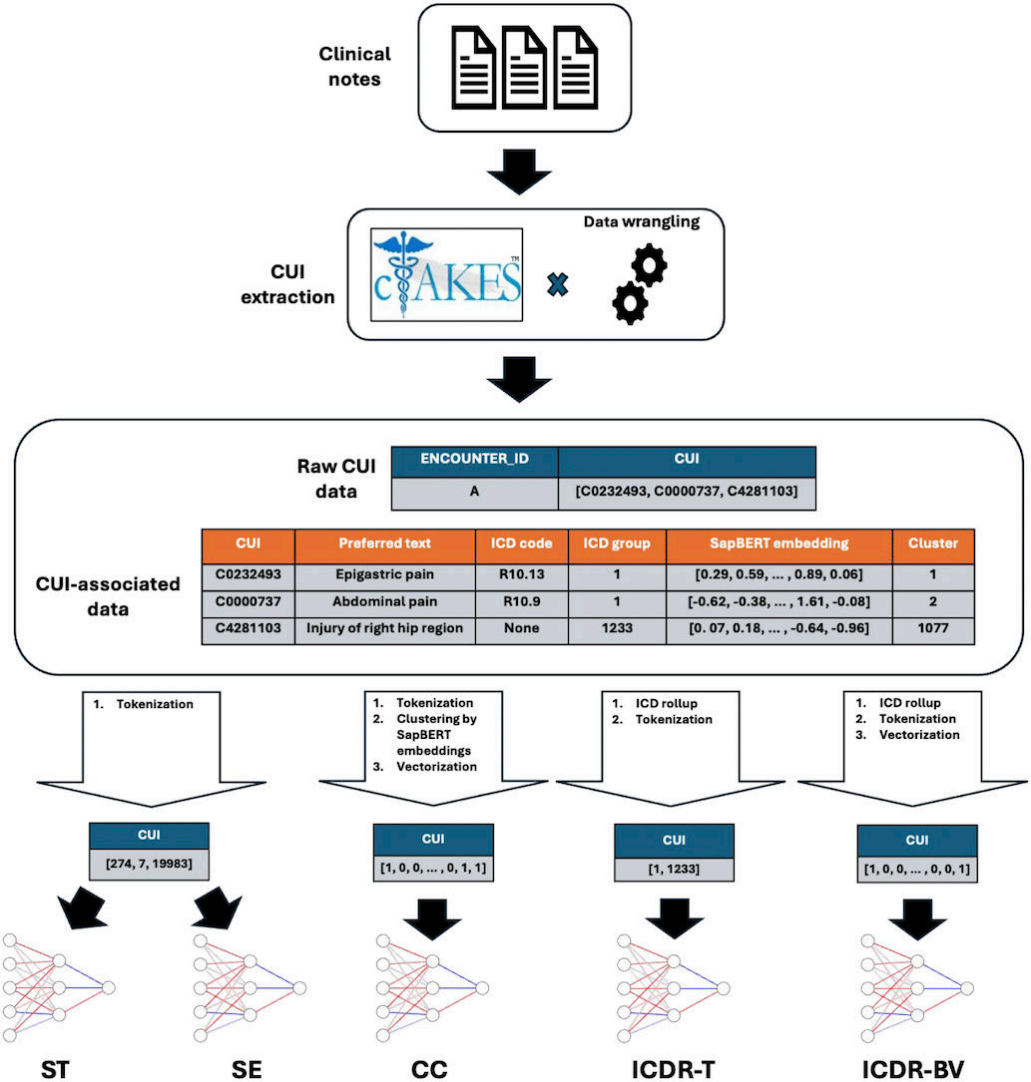
Overview of unstructured data processing for a single timestep. CUIs are extracted from clinical notes by cTAKES and assembled into a usable data structure that maps encounter ID to a list of CUIs associated with the timestep. A table of CUI-associated data is created, independent of patient data, containing preferred text, *ICD* codes, SapBERT embedding, and cluster information. The CUI-associated data is transformed according to the form required for each model, denoted by the labels beneath each neural network. The data shown are meant to illustrate the processing pipeline and do not have the exact values of patient or CUI-associated data. CC: concept unique identifier clustering using SapBERT embeddings; cTAKES: Apache Clinical Text Analysis and Knowledge Extraction System; CUI: concept unique identifier; ICD: *International Classification of Diseases*; ICDR-BV: *International Classification of Diseases* rollup using binary variables; ICDR-T: *International Classification of Diseases* rollup using tokenization; SE: concept unique identifiers as SapBERT embedding; ST: standard tokenization.

Clinical notes data were preprocessed using the Apache Clinical Text Analysis and Knowledge Extraction System (cTAKES), a tool that maps medical terms from the National Library of Medicine’s Unified Medical Language System (UMLS) to CUIs [23,24]. For example, the term “headache” maps to the CUI “C0018681.” CUIs enable us to focus on the medical terms contained within the notes and create a harmonized structure to represent notes from distinct health systems. To keep the CUI vocabularies consistent between the UC and the UW data, only CUIs present in the UC development data were kept in the UW data. Furthermore, these CUIs were subset to those appearing in at least 5 admissions in the UC data. The CUIs were timestamped with the datetimes from their clinical note of origin, then matched with the structured data by pulling forward the most recent 360 unique CUIs that appeared in the clinical notes before each structured timestep. This number was chosen due to GPU memory constraints. Deep learning models cannot operate on CUIs themselves because they are strings. Thus, we investigated various approaches for parameterizing CUIs into model-ready numerical representations (Figure 1).

#### Standard Tokenization

The first method of CUI parameterization used a Keras TextVectorization layer to tokenize a list of CUIs into a vector of integers, where there was a unique one-to-one mapping from CUI to integer. These integers get mapped to dense vectors. In total, 31,418 unique CUIs were shared between the UC and the UW data.

#### *International Classification of Diseases* Rollup Using Tokenization

In a second strategy designed to make the CUI inputs more parsimonious, we grouped CUIs capturing similar medical concepts based on the *International Classification of Diseases* (*ICD*) codes associated with them in the UMLS metathesaurus [24]. As *ICD* codes are hierarchical in nature, we used them to “roll up” CUIs whose *ICD* codes share the first 3 characters into a unique *ICD* category. All CUIs without an associated *ICD* code were assigned to the same category. Mirroring the ST approach, each grouping of CUIs by *ICD* codes was then mapped to a unique integer. The *ICD* rollup groupings were first computed on the UC CUI data and then applied to the UW CUI data. Compared to the ST approach, this process reduced the number of unique tokens from 31,418 to 1232.

#### *ICD* Rollup Using Binary Variables

Using the tokenized CUI data created in the *International Classification of Diseases* rollup using tokenization (ICDR-T) approach, we converted each vector of tokens to a 1232-dimensional sparse binary vector, where each index of the sparse vectors represents the presence or absence of a CUI contained by the *ICD* rollup grouping corresponding to that index.

#### CUIs as SapBERT Embeddings

**Figure 2.**
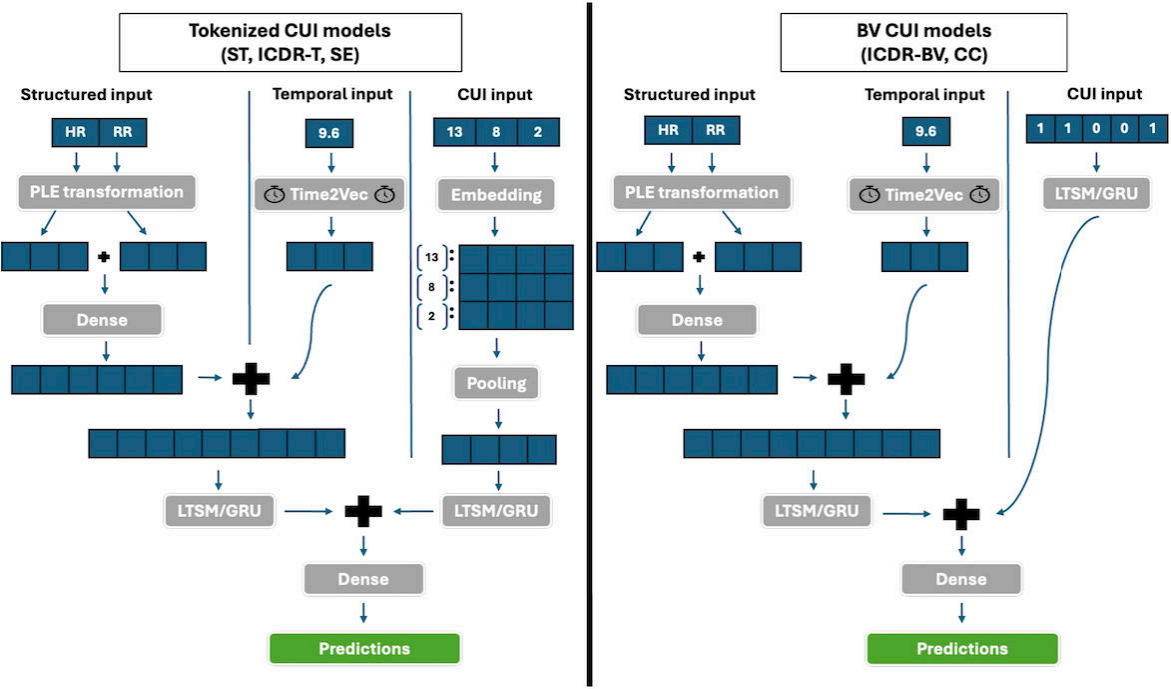
Model architectures for tokenized versus BV CUIs. Each timestep has structured, temporal, and CUI inputs. The structured and temporal inputs are transformed through a series of layers before being concatenated. Then, the joint representation of the structured and temporal inputs is concatenated with the transformed CUI input to make the final prediction. BV: binary variable; CC: concept unique identifier clustering using SapBERT embeddings; CUI: concept unique identifier; GRU: gated recurrent unit; HR: heart rate; ICDR-BV: *International Classification of Diseases* rollup using binary variables; ICDR-T: *International Classification of Diseases* rollup using tokenization; LSTM: long short-term memory; PLE: piecewise linear encoding; RR: respiratory rate; SE: concept unique identifiers as SapBERT embedding; ST: standard tokenization.

Our next approach to CUI parameterization extended our standard tokenization (ST). We extracted the preferred text strings associated with each CUI (eg, CUI: “C0000737” → preferred text: “unspecified abdominal pain”) and obtained a 768-dimensional embedding for each string using Hugging Face’s SapBERT model, a transformer model trained on UMLS medical terms [25,26]. We then generated a mapping from token → SapBERT embedding that served as the basis for a pretrained embedding matrix. When using this embedding approach in our models, the vector of tokens at each timestep is transformed into a matrix of SapBERT embeddings, where there is a one-to-one correspondence between the token value and the row of the embedding matrix that contains that token’s SapBERT embedding (Figure 2).

#### CUI Clustering Using SapBERT Embeddings

Our final approach to CUI parameterization used the CUI’s SapBERT embeddings to cluster the CUIs. First, we performed PCA on the embeddings to reduce their dimensionality from 768 to 100. Then, we calculated the pairwise cosine distances between the 100-dimensional embeddings. Lastly, we performed hierarchical clustering with a distance threshold to obtain 1077 clusters.

With these embedding clusters, we created clusters of tokenized CUIs. These token clusters enabled us to transform the vectors of tokenized CUIs from the ST CUI data into a 1077-dimensional sparse vector. Like the *International Classification of Diseases* rollup using binary variables (ICDR-BV) approach, each index of the embedding represents the presence or absence of a CUI contained within the cluster associated with that index.

### Model Development

For each CUI parameterization method, a model was developed using an intermediate fusion architecture with long short-term memory (LSTM) or gated recurrent unit (GRU) layers that learned representations of the structured, unstructured (CUI), and temporal inputs separately before combining them to learn their joint interactions. Both the number and type of recurrent layers were hyperparameters. In all models, the structured data was first passed through a fully connected layer, followed by a dropout layer. The temporal data were passed through a Time2Vec layer, a layer for vectorizing time shown to improve LSTM performance. Time2Vec represents time in a neural network by encoding temporal information to capture both periodic and nonperiodic patterns [27]. This may include, for example, events occurring on a daily or weekly basis throughout a patient encounter. The structured and temporal data were then concatenated and passed through the LSTM/GRU layers.

The CUI data were processed according to their parameterization method. For the ST and ICDR-T approaches, the tokens were transformed into randomly initialized embeddings, which were then passed through a dropout layer. The resulting embeddings were collapsed into a 1D embedding by average or max pooling, passed through a dropout layer, and then passed through LSTM/GRU layers. A similar process was also applied to the concept unique identifiers as SapBERT embedding (SE) approach, with the difference being that the tokens were mapped to their corresponding SapBERT embedding rather than to a randomly initialized one. For the methods parameterizing CUI data as sparse binary vectors (ICDR-BV and concept unique identifier clustering using SapBERT embeddings [CC]), the CUI data were passed directly through a dropout layer, then through LSTM/GRU layers.

The learned representations of the structured, temporal, and unstructured (CUI) data were fused into a single joint representation. This joint representation was passed through a final fully connected layer with a sigmoid activation function to produce the model’s predictions. A separate, structured data-only model was fit in the same manner but without the additional CUI data.

For hyperparameter optimization, we used the Keras BayesianOptimization Tuner with an 80/20 training/validation split on the model development data (UC). We ran 20 trials with early stopping if the validation area under the receiver operating characteristic curve (AUROC) did not improve by ≥0.005 for 5 consecutive epochs. Batches of size 32 were used for all models, except for the SE model, whose batch size was 16 to not exhaust GPU resources. The external validation data (UW) was not used during model development.

### Model Evaluation

Model discrimination was assessed using the area under the precision-recall curve (AUPRC) and the AUROC as primary and secondary performance metrics, respectively. AUPRC was chosen due to the low outcome prevalence in the development and validation cohorts. With a low outcome prevalence, AUROC scores can be overly optimistic, as a model that rarely predicts the outcome correctly can still have a high accuracy and low FPR. This allows models with relatively poor positive predictive values, and therefore clinical utility, to have misleadingly high AUROC scores. AUPRCs with 95% CIs were calculated using bootstrapping with 1000 iterations, where each bootstrapping iteration was done using a sample size equal to 20% of the population size of each subgroup. AUROCs with 95% CIs were calculated using R (version 4.4.0; R Foundation) with the *pROC* (version 1.18.5) package. Sensitivity, specificity, positive predictive value, and negative predictive value were calculated across a range of cut points.

Subgroup analyses were conducted to assess model performance across sex, race, ethnicity, and age. Differences in patient characteristics were assessed using chi-squared tests for categorical variables and Mann-Whitney U tests for age.

Initial data cleaning was carried out using Stata (version 16.1; StataCorp). Preprocessing, descriptive analysis, and model development were carried out using Python (version 3.9.18; Python Software Foundation), with data analysis and machine learning libraries such as TensorFlow (version 2.12.0; Google LLC), Keras (version 2.12.0; Google LLC), KerasTuner (version 1.3.5; Google LLC), scikit-learn (version 1.2.0), SciPy (version, 1.10.1), and Pandas (version 1.5.3; NumFOCUS, Inc).

### Model Explainability With Integrated Gradients

To demonstrate the potential utility of using CUIs in clinically deployed models, we applied an explainability method called integrated gradients (IGs) to our ST model [28]. IG enabled us to determine which CUIs were most influential for the model’s predictions. For a given input x at a single timestep, IG works by calculating the average gradient relative to the model’s output as the input changes from its baseline values. The exact method for calculating the attribution score for input x can be found in the original paper [28]. For computational efficiency, we can approximate the exact method by using a Riemann sum approximation of the integral. Again, the exact formula for this calculation can be found in the original paper [28].

We applied the approximate IG method on a balanced sample of 5000 patients from the external validation (UW) cohort to obtain attribution scores for each dimension of each CUI embedding in the model inputs. As this IG method only gives attribution scores at the timestep level, we needed to derive a method for calculating a global attribution score that captured the relative importance of all CUIs used in the model. To accomplish this for a given CUI, we summed the attribution scores across all the CUI’s embedding dimensions, timesteps, and encounters to get a single global attribution score. This process was carried out for each CUI. To adjust for differences in CUI frequency, we multiplied the global attribution score for each CUI by its inverse document frequency (IDF). The IDF of each CUI is given by:


(1) $$IDF_{CUI_{j}}=\log\frac{N}{df_{CUI_{j}}}$$

where N is the number of encounters in the sample (n=5000) and $df_{CUI_{j}}$ is the number of encounters in which CUI j appears. Thus, if we define x to be the CUI input, $T_{n}$ to be the number of timesteps in encounter n, D to be the number of dimensions in the embedding of CUI j, and $IDF_{CUI_{j}}$ to be the IDF of CUI j, then the final equation for our CUI importance score is given by:


(2) $$\begin{array}{r} {\text{ImportanceScore}}_{CUI_{j}}\\ =\sum_{n=1}^{N}\sum_{t=1}^{T_{n}}\sum_{i=1}^{D}\text{IntegratedGrads}{\;}_{i}^{\left(approx\right)}(x)\cdot IDF_{CUI_{j}} \end{array}$$

### Ethical Considerations

Ethical approval of this work was given by the UW Minimal Risk Research Institutional Review Board (#2019‐1258) and the UC Biological Sciences Division Institutional Review Board (#18‐0447). Informed consent was not obtained, as the study was retrospective and the study protocol was granted a waiver from informed consent. Additionally, all patient data were deidentified before use. No compensation was provided to patients from whom data were used.

## Results

### Cohort Characteristics

The model development cohort (UC) included 284,302 patients, with 14,954 (5.26%) deteriorating during their hospitalization. The external validation cohort (UW) included 248,055 patients, with 11,327 (4.57%) deteriorating during their hospitalization. Across both cohorts (UC and UW), patients who experienced deterioration were more likely to be male (14,371/26,281, 54.68%) than female (11,910/26,281, 45.32%), older, and have a greater median length of stay than those who did not experience deterioration (Table S2 in Multimedia Appendix 1). Other demographic variables were statistically significant between those who did and did not experience deterioration, but the magnitudes of these differences were small.

Patient characteristics also varied between sites (Table 1). The model development site (UC) had a higher proportion of females (164,615/284,302, 57.9% vs 119,959/248,055, 48.36%) and a lower proportion of White patients (111,797/284,302, 39.32% vs n=225,212/248,055, 90.79%). Other differences were statistically significant due to the large sample size, but were numerically small.

**Table 1.** Comparison of patient characteristics between the model development (UC^a^; n=284,302) and external validation (UW^b^; n=248,055) cohorts.

Characteristic		UC	UW	*P* value
Length of stay (hours), median (IQR)		74.8 (102.6)	79.6 (98.7)	<.001
Death or ICU^c^ transfer, n (%)		14,954 (5.3)	11,327 (4.6)	<.001
Ward to ICU transfer, n (%)		13,925 (4.9)	9200 (3.7)	<.001
In-hospital death, n (%)		1886 (0.7)	2760 (1.1)	<.001
Age (years), median (IQR)		56 (29)	59 (30)	<.001
Sex, n (**%**)				
	Female	164,615 (57.9)	119,959 (48.4)	<.001
Race, n (**%**)				
	White	111,797 (39.3)	225,212 (90.8)	<.001
	Black	147,856 (52)	13,735 (5.5)	<.001
	Asian or Mideast Indian	6645 (2.3)	3810 (1.5)	<.001
Ethnicity, n (**%**)				
	Hispanic or Latino	14,931 (5.3)	6551 (2.6)	<.001
Age (years), n (%)				
	18‐33	53,538 (18.8)	31,121 (12.5)	<.001
	34‐48	50,636 (17.8)	42,168 (17)	<.001
	49‐64	86,861 (30.6)	86,333 (34.8)	.21
	65‐78	68,134 (24)	61,157 (24.7)	<.001
	>79	25,133 (8.8)	27,276 (11)	<.001

a UC: University of Chicago.

b UW: University of Wisconsin-Madison.

c ICU: intensive care unit.

### Model Performance

**Figure 3.**
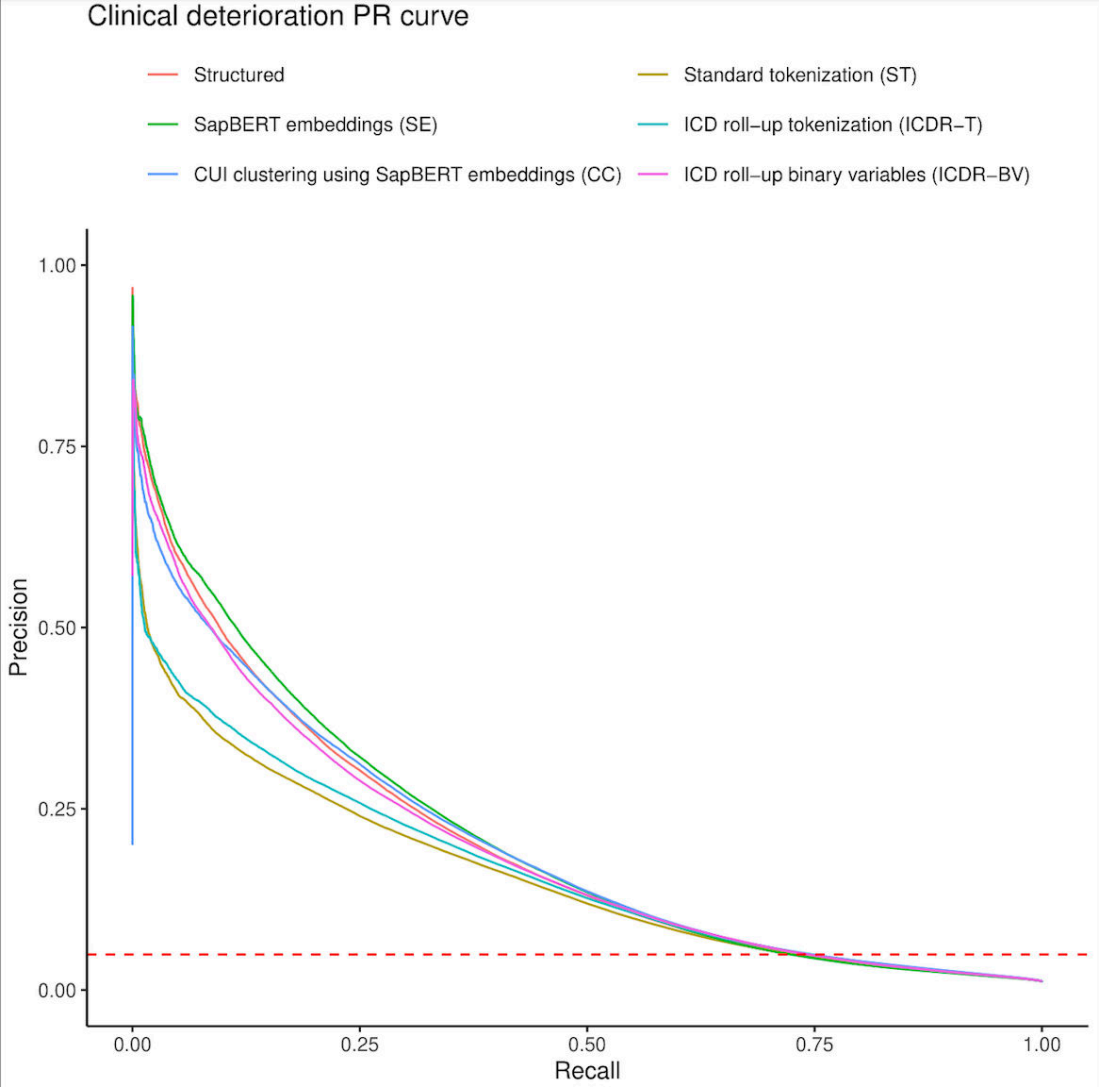
AUPRCs for the multimodal and structured-only models in the external validation cohort. The dashed red line indicates the baseline outcome prevalence of 4.9%. AUPRC: area under the precision-recall curves; CUI: concept unique identifier; ICD: *International Classification of Diseases*; PR: precision-recall.

The AUPRC scores with their 95% CI for predicting clinical deterioration are presented for each model in Tables S3 and S4 in Multimedia Appendix 1. The SE model had the highest AUPRC (0.208), followed by the structured-only (0.199) and CC (0.199) models, ICDR-BV (0.194), ICDR-T (0.166), and ST (0.158; Figure 3). The structured-only and CC models achieved the highest AUROC (0.870), followed by ICDR-T (0.867), ICDR-BV (0.866), ST (0.860), and SE (0.859; Tables2  3).

Some variation of model performance existed across subgroups. Across all models, the AUPRC scores for Asian/Mideast Indian patients were the greatest on average (0.226, SD 0.019), while they were the lowest on average for patients between the ages of 18‐30 years (0.159, SD 0.010). AUROC scores were more tightly clustered than AUPRC scores across subgroups. Still, the average AUROC score across models was greatest for patients in the age range of 34‐48 years (0.875, SD 0.010), while it was the lowest for patients aged ≥79 years (0.848, SD 0.010). The SE model had the highest performance across all subgroups for AUPRC, while the best-performing model differed across subgroups for AUROC.

Table S5 in Multimedia Appendix 1 depicts the sensitivity, specificity, positive, and negative predictive values for models assessed along a range of probability cutoffs corresponding to the highest-risk 15%, 10%, 5%, and 1% of observations being flagged for clinical deterioration. At the 5% and 10% cutoffs, the CC model achieved the greatest positive predictive values (12.53% and 7.66%, respectively) and sensitivities (52.15% and 63.79%, respectively). At the 15% cutoff, the ICDR-T, CC, and ICDR-BV models tied for the highest positive predictive value at 5.67%, while their sensitivities were 70.95%, 70.92%, and 70.86%, respectively. The average positive predictive values across models at the 5% and 15% cutoffs were 12.30% and 5.63%, respectively, while the average sensitivities were 51.23% and 70.27%.

Regarding model calibration, Table S6 in Multimedia Appendix 1 shows the Brier scores for all models on the external validation (UW) cohort. The ICDR-BV, SE, and CC models had the best and lowest Brier scores (0.011), while the ST and ICDR-T models scored slightly worse (0.012; Table S6 in Multimedia Appendix 1).

After applying our modified IG approach to our ST model, some most important CUIs corresponded to the terms “NPO [nil per os] – nothing by mouth,” “chemotherapy,” “transplanted tissue,” and “dialysis procedure” (Table S7 in Multimedia Appendix 1). The complete table of high-importance CUIs is presented in Table S7 in Multimedia Appendix 1.

**Table 2.** Model AUROCs^a^ for the ST^b^, ICDR-T^c^, and ICDR-BV^d^ models on the external validation cohort (UW^e^) across subgroups.^f^

Subgroup		ST, AUROC (95% CI)	ICDR-T, AUROC (95% CI)	ICDR-BV, AUROC (95% CI)
All		0.86 (0.859‐0.861)	0.867 (0.866‐0.868)	0.866 (0.865‐0.866)
Sex				
	Female	0.869 (0.868‐0.87)	0.873 (0.871‐0.874)	0.873 (0.872‐0.874)
Race				
	White	0.859 (0.858‐0.86)	0.866 (0.865‐0.867)	0.865 (0.864‐0.865)
	Black	0.869 (0.866‐0.872)	0.87 (0.866‐0.874)	0.874 (0.871‐0.878)
	Asian or Mideast Indian	0.866 (0.86‐0.873)	0.875 (0.869‐0.882)^f^	0.873 (0.867‐0.88)
Ethnicity				
	Hispanic or Latino	0.849 (0.844‐0.854)	0.853 (0.847‐0.858)	0.858 (0.853‐0.863)
Age (years)				
	18‐33	0.867 (0.864‐0.87)	0.863 (0.86‐0.866)	0.863 (0.86‐0.866)
	34‐48	0.873 (0.871‐0.875)	0.876 (0.875‐0.878)	0.876 (0.874‐0.878)
	49‐64	0.862 (0.861‐0.864)	0.872 (0.871‐0.874)	0.87 (0.869‐0.871)
	65‐78	0.846 (0.844‐0.847)	0.854 (0.852‐0.855)	0.853 (0.852‐0.855)
	>79	0.845 (0.843‐0.847)	0.851 (0.849‐0.853)	0.85 (0.848‐0.852)

a AUROC: area under the receiver operating characteristic curve.

b ST: standard tokenization.

c ICDR-T: *International Classification of Diseases* rollup using tokenization.

d ICDR-BV: *International Classification of Diseases* rollup using binary variables.

e UW: University of Wisconsin-Madison.

f This is the best score for each subgroup between Tables2  3.

**Table 3.** Model AUROCs^a^ for the structured, SE^b^, and CC^c^ models on the external validation cohort (UW^d^) across subgroups.

Subgroup		Structured, AUROC (95% CI)	SE, AUROC (95% CI)	CC, AUROC (95% CI)
All		0.87 (0.869‐0.871)^*e*^	0.859 (0.858‐0.859)	0.87 (0.869‐0.871)^e^
Sex				
	Female	0.861 (0.86‐0.862)	0.867 (0.866‐0.868)	0.875 (0.874‐0.876)^e^
Race				
	White	0.87 (0.866‐0.873)^e^	0.857 (0.856‐0.858)	0.869 (0.868‐0.87)
	Black	0.873 (0.867‐0.879)	0.871 (0.868‐0.875)	0.881 (0.878‐0.884)^e^
	Asian or Mideast Indian	0.844 (0.839‐0.85)	0.871 (0.865‐0.878)	0.872 (0.866‐0.879)
Ethnicity				
	Hispanic or Latino	0.862 (0.859‐0.865)^e^	0.852 (0.847‐0.858)	0.858 (0.853‐0.863)
Age (years)				
	18‐33	0.871 (0.869‐0.873)^e^	0.854 (0.851‐0.857)	0.871 (0.868‐0.874)^e^
	34‐48	0.868 (0.867‐0.869)	0.87 (0.868‐0.872)	0.882 (0.881‐0.884)^e^
	49‐64	0.847 (0.846‐0.849)	0.864 (0.863‐0.866)	0.875 (0.874‐0.876)^e^
	65‐78	0.846 (0.844‐0.848)	0.843 (0.841‐0.844)	0.857 (0.856‐0.858)^e^
	>79	0.862 (0.861‐0.863)^e^	0.844 (0.842‐0.846)	0.853 (0.851‐0.855)

a AUROC: area under the receiver operating characteristic curve.

b SE: concept unique identifiers as SapBERT embedding.

c CC: concept unique identifier clustering using SapBERT embeddings.

d UW: University of Wisconsin-Madison.

e This is the best score for each subgroup between Tables2  3.

## Discussion

### Principal Findings

In this study, we compared 5 methods of CUI parameterization for use in multimodal deep learning models, as well as a structured-only model to predict clinical deterioration in non-ICU inpatients. All models demonstrated strong performance and were well calibrated, and discrimination was similar overall, especially with the AUROC metric. We found no meaningful differences between models that used tokenized+embedded CUI data, such as the SE, ICDR-T, and ST models, versus those that included CUI data as a vector of binary variables, such as ICDR-BV and CC models. However, our method, using SapBERT embeddings to represent CUIs (SE), achieved the highest AUPRC overall and across all subgroups. Importantly, this performance was only slightly better than the structured data-only model. Our results suggest that the addition of medical terms as CUIs in multimodal models does not meaningfully improve performance beyond models using structured data alone. However, the inclusion of CUIs in deterioration models may provide clinical utility irrespective of improving a model’s predictive ability.

As deep neural networks such as those featured in our paper are difficult to interpret, we cannot say with confidence why CUI models did not outperform the structured-only model. Nonetheless, several potential causes may be contributing to the similarity in model performance. One potential cause is that the CUIs do not add more useful information to the models. That is, the CUIs do not fill in any gaps in the structured EHR data that enables the models to make more accurate predictions. Alternatively, CUIs themselves may be adding more noise than signal to the models or could be contributing to overfitting. When including CUIs in multimodal models, our results suggest a benefit in restricting the number of CUI-related variables in the input space. The CC, ICDR-T, and ICDR-BV models restrict the number of CUI-related variables in their unstructured input from 31,418 to 1077, 1232, and 1232, respectively, while the ST and SE models use the full 31,418. The models restricting the CUI-related variable space achieved higher AUROCs, positive predictive values, and sensitivities at the 15% cutoff relative to SE and ST models. However, this pattern does not hold as strongly for AUPRC scores. The ST model, which does not restrict the number of CUI-related variables, achieved a relatively low AUPRC, as it did for AUROC, but the SE model, which achieved the lowest AUROC, achieved the highest AUPRC score. When considering all performance metrics together, the CC model may have a slight edge above the other CUI models, as it achieved the highest AUROC, positive predictive values, and sensitivities at the 5% cutoff, the highest positive predictive value and sensitivity (tie) at the 10% cutoff, the highest positive predictive value (tie) at the 15% cutoff, and the second highest AUPRC. However, all differences in performance metrics were small. Importantly, the optimal model for clinical implementation would be based on the cutoff threshold picked that would prompt specific clinical actions or alerts needed for clinical practice. As such, selecting models with the highest positive predictive values will lead to the fewest number of false alarms, as their rate of false positives will be the lowest. With this in mind, the CC model, as well as the other models that restrict the number of CUI-related variables (ICDR-T and ICDR-BV), may be preferable in a clinical context to models that do not (SE and ST).

We also found that the structured-only model performed just as well and often better than most of the models that used CUIs, but that implementing explainability techniques alongside multimodal models could help confirm a clinician’s intuition and support in decision-making. The application of our modified IG method to the ST model provides a proof of concept for a deep learning model that could provide a clinician with high-value terms from a patient’s notes in addition to making strong predictions of their overall deterioration risk. Still, it is important to note that we only applied our IG method to the ST model because it was the simplest multimodal model, so it lends itself the best to explainability techniques. More sophisticated models such as the SE, CC, ICDR-T, and ICDR-BV models, would require more careful application and interpretation of explainability techniques to garner clinically useful results. Thus, the higher complexity of implementing and interpreting the CUI models must be weighed against the additional clinical context information that could be provided by these models (eg, using explainable artificial intelligence techniques) when considering whether to use these models in practice.

To our knowledge, this is the first paper to investigate multimodal integration of information from structured and unstructured data in deep learning models to predict clinical deterioration in ward patients. A recent systematic review by van der Vegt et al [29] examined the current use of artificial intelligence for predicting clinical deterioration. They found that while many groups have developed promising machine learning models using methods such as logistic regression, Extreme Gradient Boosting, and random forests, few have developed deep learning models. Deep learning models have, on average, outperformed nondeep learning models [30-32]. Additionally, groups have developed deep learning models for predicting deterioration using ICU patients, patients from the emergency department, or patients with COVID-19 [10,21,33-36]. However, few have developed models using ward patients [11,30]. Fewer still have used multiple input modalities, none of which focused on ward deterioration [37]. As predicting clinical deterioration early and accurately leads to improved outcomes, efforts to increase model accuracy such as ours could lead to enhanced detection and decrease false alarms. Furthermore, the incorporation of information from clinical notes could provide additional context for clinicians related to factors that increase a patient’s risk of the event.

Our methods also demonstrate the ability to make effective and fair predictions in ward patients. Existing papers developing deep learning models in ward patients did not provide analyses on the performance of their models across patient subgroups, which is critical given the diverse nature of ward patients [11,30,37]. Training machine learning models on observational data for use in clinical settings can be biased in favor of populations for whom there is more training data. This raises concerns about the ethics of deploying such unfair models for use in a clinical setting. However, the development (UC) and validation (UW) cohorts contained large demographic differences, but the performance differences of our models across demographic groups were small. Our models obtained strong performance on patients with diverse demographics coming from variable hospital settings. AUROCs were slightly higher on average for patients aged 34‐48 years when compared to other subgroups, and slightly worse for patients near the tail ends of the age distribution (18‐33 and ≥79 years). Furthermore, the model discrimination was superior for Black patients when compared to other racial subgroups. This might be explained by the high proportion (n=147,856, 52%) of Black patients in the development (UC) cohort. Additionally, the AUPRC scores for patients aged 18‐33 years were uniformly the lowest across all models. A likely cause for this may be that the proportion (7.3%) of these patients was lower than any other subgroup.

Our study has several strengths. First, incorporating multimodal data into deep learning models is rare within the ward deterioration literature, as most groups who have developed deep learning models have used exclusively structured EHR data [10,29-31,34,35,38-40]. Our study provides a diverse set of methods to inspire further exploration of multimodal modeling of clinical deterioration. Second, each CUI parameterization method and its associated model’s performance were externally validated in a separate health system. Large discrepancies existed between the development and external validation cohorts, and note-taking practices can differ greatly between hospital systems, which could increase variability in the CUIs for similar patients [41-43]. Despite these challenges, all models exhibited strong performance, suggesting the robustness and generalizability of our methods to diverse settings.

### Limitations

Our study is limited in that we did not explore all possible CUI parameterization methods, and we only focused on predicting a single outcome. Additionally, this study did not use the raw text that constitutes clinical notes. Thus, we cannot draw strong conclusions about the overall utility of clinical notes in clinical prediction models. We also recognize that implementing deep learning models with CUI inputs can be practically challenging. However, we have provided guidance and demonstrated the feasibility of real-time support for natural language processing tools in our recent work [44,45]. Finally, this was a retrospective study, and the usefulness of a clinical prediction model needs to be tested in a prospective clinical trial, which is outside the scope of this work.

### Conclusions

In conclusion, we demonstrated the feasibility of combining structured and unstructured EHR data in deep learning models to predict clinical deterioration. While the best-performing model varied by metric, those whose method of CUI parameterization used SapBERT embeddings may have an edge over others, as the SE model performed best concerning our primary metric (AUPRC), while the CC model seemed to perform best when considering all metrics in aggregate, achieving the highest AUROC, second highest AUPRC, and highest positive predictive value at multiple cutoffs. However, the structured-only model performed similarly to the models that included CUIs, suggesting that the marginal gains in performance may not outweigh the increased complexity and difficulties in interpreting CUI models. This work adds to the field of predicting clinical deterioration, which has historically only focused on structured data.

## Supplementary material

10.2196/75340 Multimedia Appendix 1 Revised supplementary material.

## Abbreviations

AUPRC: area under the precision-recall curve
AUROC: area under the receiver operating characteristic curve
CC: concept unique identifier clustering using SapBERT embedding
cTAKES: Clinical Text Analysis and Knowledge Extraction System
CUI: concept unique identifier
EHR: electronic health record
FPR: false positive rate
GRU: gated recurrent unit
*ICD*: *International Classification of Diseases*
ICDR-BV: *International Classification of Diseases* rollup using binary variables
ICDR-T: *International Classification of Diseases* rollup using tokenization
ICU: intensive care unit
IDF: inverse document frequency
IG: integrated gradient
LSTM: long short-term memory
SE: concept unique identifiers as SapBERT embedding
UC: University of Chicago
UMLS: Unified Medical Language System
UW: University of Wisconsin-Madison
