# Supplementary material for: Comparison of Multimodal Deep Learning Approaches for Predicting Clinical Deterioration in Ward Patients: Observational Cohort Study
Source: J Med Internet Res. 2025 Jun 11;27:e75340. doi: 10.2196/75340 (PMC12176310; doi:10.2196/75340)
Supplement: Multimedia Appendix 1 [file jmir-v27-e75340-s001.docx]

**Table S1.** Predictor variables used in the models.

| Measurements | | Variable Type |
| --- | --- | --- |
|  |  |  |
| **Demographics** | |  |
|  | Age | Continuous |
| **Vital signs** | |  |
|  | Temperature (C°), Heart Rate, Respiratory Rate, Systolic Blood Pressure (SBP), Diastolic Blood Pressure (DBP), O2 Saturation, Fraction of Inspired Oxygen (FiO2), AVPU, Disorientation (yes/no) | Continuous |
| **Laboratory values** | |  |
|  | Basic Metabolic Panel [BMP]: Sodium, Chloride, Potassium, Bicarbonate (CO2), Anion Gap, Glucose, Calcium, Blood Urea Nitrogen (BUN), Serum Creatinine (SCr), Phosphate | Continuous |
|  | Liver Function Test [LFT]: Total Protein, Albumin, Total Bilirubin, Aspartate Aminotransferase (AST/SGOT), Alkaline Phosphatase | Continuous |
|  | Complete Blood Count [CBC]: White Blood Cells (WBC), Hemoglobin, Platelet Count, Bands, Eosinophils, Lymphocytes, Monocytes, Neutrophils | Continuous |
|  | Blood Gas test: Arterial pH, Venous pH, Arterial Partial Pressure of Oxygen, Arterial Partial Pressure of Carbon Dioxide, Venous Partial Pressure of Carbon Dioxide | Continuous |
|  | Other labs: Lactate, Magnesium, Lipase, International Normalized Ratio (INR), Mean Corpuscular Volume (MCV), Partial Thromboplastin Time (PTT), and Red Cell Distribution Width (RDW) | Continuous |
| **Nurse Documentation** | |  |
|  | Braden Scale (Activity, Friction and Shear, Mobility, Moisture, Nutrition, Sensory Perception, Total Score), Body Mass Index | Continuous |
| **Length of stay** | |  |
|  | Hours since admission until the current time point | Continuous |
| **Time of Day** | |  |
|  | Hours since midnight of the current day | Continuous |
| **Urinary Output** | |  |
|  | Sum of Urine Output over the last 24 hours | Continuous |

**Table S2.** Comparison of patient characteristics between those with (N=26,281) and without clinical deterioration (N=506,076) in the study cohort.^a^

| Characteristic | | Deterioration | No Deterioration | *P* |
| --- | --- | --- | --- | --- |
|  |  |  |  |  |
| Length of stay, hours, median (IQR) | | 217.5 (294.6) | 74.2 (93.7) | < .001 |
| Age, median (IQR) | | 63 (21) | 57 (27) | < .001 |
| **Sex, n (%)** | |  |  |  |
|  | Female, n (%) | 11,910 (45.3) | 272,664 (53.9) | < .001 |
| **Race, n (%)** | |  |  |  |
|  | White | 16,320 (62.1) | 320,689 (63.4) | < .001 |
|  | Black | 8,231 (31.3) | 153,360 (30.3) | < .001 |
|  | Asian/Mideast Indian | 500 (1.9) | 9,955 (2.0) | < .001 |
| **Ethnicity, n (%)** | |  |  |  |
|  | Hispanic/Latino | 1,010 (3.8) | 20,472 (4.0) | < .001 |
| **Age, n (%)** | |  |  |  |
|  | 18-33 | 1,924 (7.3) | 82,735 (16.3) | < .001 |
|  | 34-48 | 3,238 (12.3) | 89,566 (17.7) | < .001 |
|  | 49-64 | 9,194 (35.0) | 164,000 (32.4) | < .001 |
|  | 65-78 | 8,298 (31.6) | 120,993 (23.9) | < .001 |
|  | ≥ 79 | 3,627 (13.8) | 48,782 (9.6) | < .001 |

^a^Abbreviations: IQR = inter-quartile range.

**Table S3.** Model AUPRCs for ST, ICRD-T, and ICDR-BV models on external validation cohort (UW) across subgroups.^a,b^

| Subgroup | | ST | ICDR-T | ICDR-BV |
| --- | --- | --- | --- | --- |
|  |  | AUPRC (95% CI) | AUPRC (95% CI) | AUPRC (95% CI) |
|  |  |  |  |  |
| All | | 0.158 (0.155-0.161) | 0.166 (0.163-0.169) | 0.194 (0.191-0.198) |
| **Sex** | |  |  |  |
|  | Female | 0.159 (0.155-0.164) | 0.165 (0.160-0.170) | 0.193 (0.188-0.199) |
| **Race** | |  |  |  |
|  | White | 0.165 (0.162-0.169) | 0.192 (0.188-0.195) | 0.165 (0.162-0.169) |
|  | Black | 0.179 (0.165-0.195) | 0.209 (0.193-0.226) | 0.179 (0.165-0.195) |
|  | Asian/Mideast Indian | 0.196 (0.168-0.228) | 0.239 (0.208-0.273) | 0.196 (0.168-0.228) |
| **Ethnicity** | |  |  |  |
|  | Hispanic/Latino | 0.174 (0.154-0.197) | 0.197 (0.176-0.222) | 0.223 (0.199-0.247) |
| **Age** | |  |  |  |
|  | 18-33 | 0.130 (0.120-0.141) | 0.146 (0.135-0.158) | 0.166 (0.153-0.179) |
|  | 34-48 | 0.163 (0.154-0.172) | 0.163 (0.154-0.171) | 0.196 (0.186-0.206) |
|  | 49-64 | 0.161 (0.156-0.165) | 0.169 (0.164-0.174) | 0.203 (0.198-0.209) |
|  | 65-78 | 0.153 (0.148-0.157) | 0.165 (0.160-0.170) | 0.189 (0.184-0.195) |
|  | > 79 | 0.181 (0.172-0.190) | 0.186 (0.177-0.195) | 0.192 (0.183-0.201) |

^a^The best score for each subgroup between Tables S3 and S4 is italicized.

^b^Abbreviations: AUPRC = area under the precision-recall curve; CI = confidence interval; ST = standard tokenization; ICDR-T = ICD rollup using tokenization; ICDR-BV = ICD rollup using binary variables.

**Table S4.** Model AUPRCs for the structured, SE, and CC models on external validation cohort (UW) across subgroups.^a,b^

| Subgroup | | Structured | SE | CC |
| --- | --- | --- | --- | --- |
|  |  | AUPRC (95% CI) | AUPRC (95% CI) | AUPRC (95% CI) |
|  |  |  |  |  |
| All | | 0.199 (0.196-0.203) | *0.208 (0.204-0.211)* | 0.199 (0.195-0.202) |
| **Sex** | |  |  |  |
|  | Female | 0.197 (0.191-0.202) | *0.205 (0.200-0.211)* | 0.195 (0.189-0.200) |
| **Race** | |  |  |  |
|  | White | 0.197 (0.193-0.200) | *0.205 (0.201-0.209)* | 0.197 (0.193-0.200) |
|  | Black | 0.217 (0.200-0.234) | *0.225 (0.209-0.242)* | 0.215 (0.199-0.234) |
|  | Asian/Mideast Indian | 0.237 (0.201-0.270) | *0.244 (0.210-0.277)* | 0.252 (0.218-0.285) |
| **Ethnicity** | |  |  |  |
|  | Hispanic/Latino | 0.228 (0.204-0.255) | *0.244 (0.220-0.272)* | 0.227 (0.205-0.254) |
| **Age** | |  |  |  |
|  | 18-33 | 0.173 (0.160-0.187) | *0.174 (0.160-0.188)* | 0.165 (0.153-0.179) |
|  | 34-48 | 0.203 (0.193-0.213) | *0.214 (0.203-0.225)* | 0.201 (0.190-0.211) |
|  | 49-64 | 0.211 (0.205-0.217) | *0.223 (0.216-0.229)* | 0.215 (0.209-0.221) |
|  | 65-78 | 0.191 (0.185-0.196) | *0.199 (0.193-0.204)* | 0.189 (0.183-195) |
|  | > 79 | 0.196 (0.186-0.205) | *0.200 (0.191-0.210)* | 0.195 (0.186-0.204) |

^a^The best score for each subgroup between Tables S3 and S4 is italicized.

^b^Abbreviations: AUPRC = area under the precision-recall curve; CI = confidence interval; SE = CUIs as SapBERT embedding; CC = CUI clustering using SapBERT embeddings.

**Table S5.** Sensitivity, specificity, positive predictive value, and negative predictive value for all models in the validation cohort across predicted probability cutoffs of 15%, 10%, 5%, and 1% of observations (Obs.) predicted to have a positive outcome. ^a,b,c^

| Model | | Cutoff | Obs. Above Cutoff (%) | SENS | SPEC | PPV | NPV |
| --- | --- | --- | --- | --- | --- | --- | --- |
|  |  |  |  |  |  |  |  |
| **Cutoff = 15%** | |  |  |  |  |  |  |
|  | ICDR-T | 0.018 | 3,589,339 (15.0) | 70.95 | 85.69 | 5.67 | 99.59 |
|  | CC | 0.009 | 3,586,574 (15.0) | 70.92 | 85.70 | 5.67 | 99.59 |
|  | ICDR-BV | 0.015 | 3,586,305 (15.0) | 70.86 | 85.70 | 5.67 | 99.59 |
|  | Structured | 0.020 | 3,583,379 (15.0) | 69.91 | 85.70 | 5.60 | 99.58 |
|  | ST | 0.019 | 3,854,185 (15.0) | 69.52 | 85.70 | 5.57 | 99.57 |
|  | SE | 0.020 | 3,582,715 (15.0) | 69.48 | 85.70 | 5.57 | 99.57 |
| **Cutoff = 10%** | |  |  |  |  |  |  |
|  | CC | 0.014 | 2,391,102 (10.0) | 63.79 | 90.67 | 7.66 | 99.52 |
|  | ICDR-BV | 0.023 | 2,391,717 (10.0) | 63.76 | 90.67 | 7.65 | 99.52 |
|  | ICDR-T | 0.028 | 2,393,387 (10.0) | 63.47 | 90.65 | 7.61 | 99.51 |
|  | SE | 0.029 | 2,392,567 (10.0) | 62.91 | 90.65 | 7.55 | 99.51 |
|  | Structured | 0.031 | 2,391,071 (10.0) | 62.66 | 90.65 | 7.52 | 99.50 |
|  | ST | 0.029 | 2,390,283 (10.0) | 62.21 | 90.65 | 7.47 | 99.50 |
| **Cutoff = 5%** | |  |  |  |  |  |  |
|  | CC | 0.033 | 1,194,691 (5.0) | 52.15 | 95.58 | 12.53 | 99.40 |
|  | SE | 0.057 | 1,196,661 (5.0) | 51.86 | 95.57 | 12.44 | 99.39 |
|  | ICDR-BV | 0.047 | 1,195,276 (5.0) | 51.56 | 95.57 | 12.38 | 99.39 |
|  | ICDR-T | 0.058 | 1,196,365 (5.0) | 51.01 | 95.56 | 12.24 | 99.34 |
|  | Structured | 0.063 | 1,196,168 (5.0) | 50.88 | 95.56 | 12.21 | 99.38 |
|  | ST | 0.061 | 1,196,371 (5.0) | 49.90 | 95.55 | 11.97 | 99.37 |
| **Cutoff = 1%** | |  |  |  |  |  |  |
|  | SE | 0.25 | 239,395 (1.0) | 26.01 | 99.30 | 31.18 | 99.10 |
|  | CC | 0.19 | 239,287 (1.0) | 25.55 | 99.30 | 30.63 | 99.10 |
|  | Structured | 0.29 | 239,478 (1.0) | 25.28 | 99.29 | 30.30 | 99.10 |
|  | ICDR-BV | 0.21 | 239,362 (1.0) | 24.48 | 99.29 | 29.34 | 99.09 |
|  | ICDR-T | 0.30 | 239,391 (1.0) | 22.70 | 99.26 | 27.22 | 99.06 |
|  | ST | 0.33 | 239,409 (1.0) | 21.77 | 99.25 | 26.10 | 99.05 |

^a^Rows for each cutoff are presented in decreasing order of sensitivity.

^b^Each cutoff represents the predicted probability at which a certain percentage (1%, 5%, 10%, or 15%) of observations are flagged by the model.

^c^Abbreviations: ICDR-T = ICD rollup using tokenization; CC = CUI clustering using SapBERT embeddings; ICDR-BV = ICD rollup using binary variables; ST = standard tokenization; SE = CUIs as SapBERT embedding; SENS = sensitivity; SPEC = specificity; PPV = positive predictive value; NPV = negative predictive value.

**Table S6.** Brier scores for models on external validation (UW) (N=248,055) cohort.^a^

| Model | Brier Score |
| --- | --- |
|  |  |
| Structured | 0.011 |
| ST | 0.012 |
| ICDR-T | 0.012 |
| ICDR-BV | 0.011 |
| SE | 0.011 |
| CC | 0.011 |

^a^Abbreviations: ICDR-T = ICD rollup using tokenization; CC = CUI clustering using SapBERT embeddings; ICDR-BV = ICD rollup using binary variables; ST = standard tokenization; SE = CUIs as SapBERT embedding.

**Table S7.** Top ten high-importance CUIs in the Standard Tokenization (ST) model calculated by using a modified integrated gradients method on a balanced sample (N=5,000) of the external validation (UW) cohort.

| CUI | Importance Score | Preferred Text |
| --- | --- | --- |
|  |  |  |
| C3665472 | 59.899 | Chemotherapy |
| C0419179 | 53.993 | NPO - Nothing by mouth |
| C0011946 | 31.535 | Dialysis procedure |
| C0332835 | 27.333 | Transplanted tissue |
| C0043031 | 26.685 | Warfarin |
| C0005558 | 26.071 | Biopsy |
| C1382100 | 25.657 | Solution Dosage Form |
| C0040732 | 24.474 | Transplantation |
| C0231835 | 22.717 | Tachypnea |
| C0004238 | 22.095 | Atrial Fibrillation |
